# SIMPL data, complex procedures: a national analysis of chief resident competency in foregut and bariatric surgery

**DOI:** 10.1007/s00464-026-13083-y

**Published:** 2026-07-08

**Authors:** Tyler Dann, Jamila K. Picart, Arjun Batra, Mahesh Gupta, Austin Cornish, Jenna Kiraly, Hope T. Jackson

**Affiliations:** 1 University of Michigan Medical School, Ann Arbor, MI USA; 2 Department of Surgery, University of Michigan, Ann Arbor, MI USA; 3 Center for Surgical Training and Research, University of Michigan, Ann Arbor, MI USA; 4 Institute for Healthcare Policy and Innovation, 2800 Plymouth Road, Building 16, Ann Arbor, MI 48109 USA

**Keywords:** Foregut surgery, Bariatric surgery, Surgical education, Competency, Residency training

## Abstract

**Background:**

Advanced foregut and bariatric procedures are technically complex and require high levels of operative autonomy and competency. While bariatric surgery training pathways are well established, foregut training remains less standardized. It is unclear whether graduating general surgery residents achieve readiness for independent practice in these procedures.

**Methods:**

We performed a national retrospective analysis of System for Improving and Measuring Procedural Learning (SIMPL) evaluations of postgraduate-year 5 (PGY-5) residents from September 2015 to August 2025. Foregut procedures included fundoplication, Heller myotomy, and hiatal hernia repair; bariatric procedures included sleeve gastrectomy and Roux-en-Y gastric bypass. Outcomes included attending-rated competency and meaningful autonomy. Multivariable logistic regression was used to estimate predicted probabilities of achieving competency and autonomy in a given foregut or bariatric procedure and identify factors associated with performance.

**Results:**

A total of 1694 evaluations were analyzed (620 foregut, 1074 bariatric). Attendings rated residents as autonomous in 50.7% of bariatric cases versus 38.2% of foregut cases (*p* < 0.001), and as competent in 40.7% versus 32.4%, respectively (*p* < 0.001). Procedure-level variation was observed, with higher performance in sleeve gastrectomy and fundoplication. Residents consistently rated their autonomy and competency lower than attendings across all procedures (*p* < 0.001). Multivariable analysis demonstrated that residency program accounted for approximately one-third of variability in both competency and autonomy. Predicted probability of competency was 34.4% for foregut and 38.5% for bariatric procedures, while predicted autonomy was 44.6% and 50.7%, respectively.

**Conclusions:**

Less than half of evaluations of graduating chief residents’ performance demonstrate competency or meaningful autonomy in advanced foregut and bariatric procedures. Significant variability exists across procedure type and training programs. These findings highlight potential gaps in readiness for independent practice and support the need for standardized competency-based training and assessment in complex minimally invasive surgery.

Advanced foregut and bariatric surgery are technically demanding procedures, requiring proficiency in complex anatomy, technique, and nuanced intraoperative decision-making. However, despite parallels in complexity, the postgraduate training requirements differ substantially between these two specialties. Bariatric surgery has established pathways that include dedicated fellowship training prior to independent practice, whereas foregut specialization after general surgery residency remains less standardized, and postgraduate training pathways are optional and variable [1, 2]. This variability is notable given that practicing physicians frequently cite foregut surgery as a priority topic for continued professional education [3–5]. Meanwhile, even though bariatric fellowship training is often required for credentialing, general surgeons in rural and community settings who do not pursue fellowship still encounter patients with surgical needs who have had prior bariatric procedures. Although advanced bariatric and foregut procedures are not explicitly included within the core Entrustable Professional Activities (EPAs) defined by the American Board of Surgery, residents are nonetheless exposed to these operations during training and may encounter them in clinical practice, particularly in community settings. Expectations regarding operative competency in these procedures, especially foregut operations, remain variably defined across residency and fellowship training pathways. In the context of current surgical training characterized by evolving curricula and duty hour-constrained learning environments, it is important to better understand operative autonomy and performance in these procedures among graduating chief residents.

Over the past two decades, surgical residency training has undergone substantial structural changes; however, whether these changes have enabled graduates to continue to attain competency in complex foregut and bariatric procedures remains unknown. ACGME duty hour restrictions were implemented in 2003 and modified in 2011 in order to improve resident well-being and patient safety [6, 7]. In doing so, there have been unintended consequences such as increased resident perceptions of gaps in training and reduced preparedness for independent practice [8]. Though overall resident case numbers have largely rebounded since the initial drop from the implementation of ACGME duty hour restrictions, national operative log analysis suggests that the diversity of operative experience has narrowed over time [9]. Existing literature suggests that post-residency training is needed when procedure complexity is matched by a lack of readiness on graduation. However, it is unknown whether graduates currently graduate with competency in foregut and Bariatric procedures.

To better understand operative competency during residency training, the System for Improving and Measuring Procedural Learning (SIMPL) was developed as a validated, smartphone-based platform that enables real-time intraoperative assessment of surgical performance. SIMPL is utilized by participating institutions, primarily academic general surgery training programs in the United States, and its use is voluntary rather than mandated at the national level. Following each case, supervising faculty complete evaluations of operative performance and autonomy, while residents provide parallel self-assessments of their performance. These evaluations provide a national dataset for examining operative competency and progression toward independent practice.

In order to further characterize chief resident performance in foregut and bariatric surgery, we performed a national retrospective analysis of SIMPL evaluations of clinical postgraduate-year (PGY)-5 performance of advanced foregut and bariatric procedures. We sought to assess chief residents’ preparedness for independent practice in these procedures and identify potential training gaps.

## Methods

### Study design and cohort selection

We collected operative case log data and evaluations for foregut and bariatric procedures performed by clinical PGY-5 general surgery residents from the SIMPL platform logged between September 2015 and August 2025 [10]. Data from the SIMPL platform are collected electronically from subscribing institutions via smartphone application. The application is designed to facilitate the timely postoperative logging of resident and attending assessments of resident performance, resident autonomy, and case complexity. This study was reviewed and declared exempt by the University of Michigan Institutional Review Board.

### Data source

A SIMPL evaluation comprises three elements rated by the trainee and attending physician in the case: resident performance, resident autonomy, and case complexity. The evaluation is initiated in the app, either by the trainee or the attending surgeon, with a concomitant invitation sent to the alternative party. These evaluations are case-based and are initiated following completion of a procedure. As such, not all operative cases are captured within the platform and case selection is influenced by user choice and institutional participation patterns rather than a standardized sampling process. Performance is rated on a 5-point scale: unprepared/critical deficiency, unfamiliar with procedure, intermediate, practice ready, or exceptional. Resident autonomy is assessed using a 4-point scale, modeled after the Zwisch scale: show and tell, active help, passive help, or supervision only [11]. Case complexity is rated on a 3-point scale: low, medium, or high.

Consistent with prior studies, in our analysis, the autonomy rating was dichotomized into “meaningful autonomy,” defined as ratings of passive help or supervision only, and “not meaningful autonomy,” defined as ratings of show and tell or active help [12]. The performance rating was dichotomized into “not competent,” defined as unprepared/critical deficiency, unfamiliar with procedure, or intermediate, or “competent,” defined as practice ready or exceptional.

Analyses were conducted at two levels. First, agreement between resident and attending ratings was evaluated using the original SIMPL ordinal scale for performance and autonomy prior to dichotomization. To define readiness for independent practice, this ordinal scale was dichotomized into clinically meaningful definitions of competency and autonomy.

Agreement between the resident and attending faculty for each procedure was evaluated for all three evaluation elements. Agreement was defined as both trainee and faculty attending rating each element at the same scale level prior to dichotomization. Underestimation is defined as the resident rater providing an evaluation lower than that of the faculty attending evaluator. Overestimation is defined as the resident rater providing an evaluation higher than that of the attending evaluator.

### Procedure selection

In this study, we included SIMPL ratings for foregut and bariatric procedures. Foregut procedures included in this study were gastric fundoplication (with or without gastroplasty), Heller myotomy (with or without fundoplasty), and hiatal hernia repair (including transhiatal and transthoracic approaches). Bariatric procedures included in this study were Roux-en-Y gastric bypass (RYGB) and sleeve gastrectomy (SG). Cases completed via laparoscopic, robotic, and open approaches were included in this study.

### Statistical analysis

Descriptive statistics were performed to understand the frequency and distribution of trainees completing evaluations for each procedure cohort, resident ratings, attending ratings, and rating agreement. Categorical variables were compared using chi-square tests and continuous variables were compared using *t* tests.

We then performed multivariable logistic regression to model the probability of being rated as competent by the attending rater. Covariates included in the model were case complexity, residency program, and time within the academic year (first half vs. second half). Our secondary outcome of interest was the probability of being rated as having meaningful autonomy by the attending rater. A similar regression model was used for evaluation of the secondary outcome. To quantify inter-institutional variability, we estimated an intraclass correlation coefficient (ICC) at the residency program level using multilevel logistic regression, where ICC values reflected the proportion of outcome variability attributable to residency program differences. All statistical analyses were performed using STATA SE 19.5 (StataCorp LLC), with a two-sided significance threshold of 5% for all hypothesis testing.

## Results

### Procedure type and surgical approach

In this study, 1694 SIMPL PGY-5 evaluations from 95 participating residency programs for foregut and bariatric procedures were analyzed. The mean number of SIMPL evaluations was 3.6 per trainee, with a median of 2 evaluations (interquartile range 1–4), reflecting a right-skewed distribution of assessment frequency across residents. Across the 95 participating residency programs, the mean number of evaluations per program was 46.7, with a median of 19 (interquartile range 7–53). Evaluations were categorized by procedure category: 620 (36.6%) foregut and 1074 (63.4%) bariatric. Among the foregut procedures, hiatal hernia repairs were the most represented, with 459 (74%). Among the bariatric procedures, sleeve gastrectomies were the most represented, with 664 (61.8%) included. Most bariatric procedures were performed using a laparoscopic approach (85.1%), whereas most foregut procedures were performed using an open approach (75.8%) (Table 1).

**Table 1:** Characteristics of foregut and bariatric procedures evaluated using the SIMPL assessment platform

Characteristic	Foregut (n = 620)	Bariatric (*n* = 1074)	Total (*n* = 1694)	*p*-value
Procedure type				< 0.001
Gastric fundoplication	103 (16.6%)	–	103 (6.1%)	
Heller myotomy	53 (8.5%)	–	53 (3.1%)	
Hiatal hernia repair	459 (74.0%)	–	459 (27.1%)	
Roux-en-Y gastric bypass	–	410 (38.2%)	410 (24.2%)	
Sleeve gastrectomy	–	664 (61.8%)	664 (39.2%)	
Operative approach				< 0.001
Laparoscopic	150 (24.2%)	914 (85.1%)	1064 (62.8%)	
Robotic	0 (0.0%)	150 (14.0%)	150 (8.9%)	
Open	470 (75.8%)	10 (0.9%)	480 (28.3%)	

Distribution of 1694 SIMPL evaluations of postgraduate-year 5 (PGY-5) residents performing foregut and bariatric procedures. Procedures are categorized by type and operative approach. Values are presented as the number (percentage) of evaluations within each category. Differences between foregut and bariatric procedures were assessed using chi-square testing

### Attending-rated autonomy and competency

**Fig. 1:**
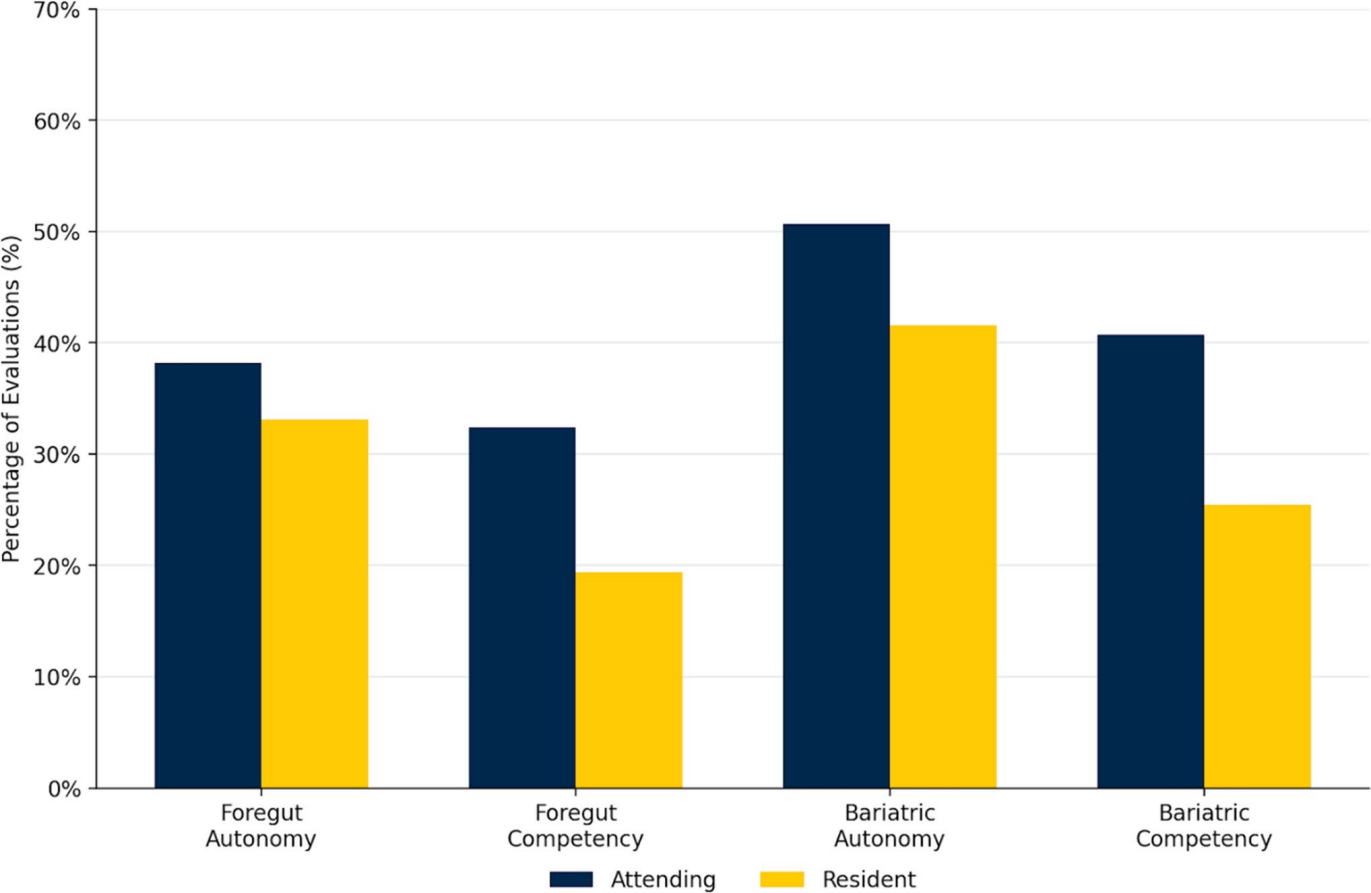
Attending and resident ratings of autonomy and competency by procedure category Comparison of attending and resident ratings of operative autonomy and competency for foregut and bariatric procedures. Bars represent the percentage of evaluations in which residents were rated as achieving meaningful autonomy or competent performance in foregut and bariatric procedures by attending surgeons or by resident self-assessment

Attendings rated bariatric procedures significantly higher than foregut procedures for both autonomy and competency. Attending-rated autonomy was 50.7% for bariatric procedures compared with 38.2% for foregut procedures (*p* < 0.001). Similarly, attending-rated competency was 40.7% for bariatric procedures and 32.4% for foregut procedures (*p* < 0.001). Overall, less than half of attending evaluations classified residents as competent or autonomous for either procedure category (Fig. 1).

**Fig. 2:**
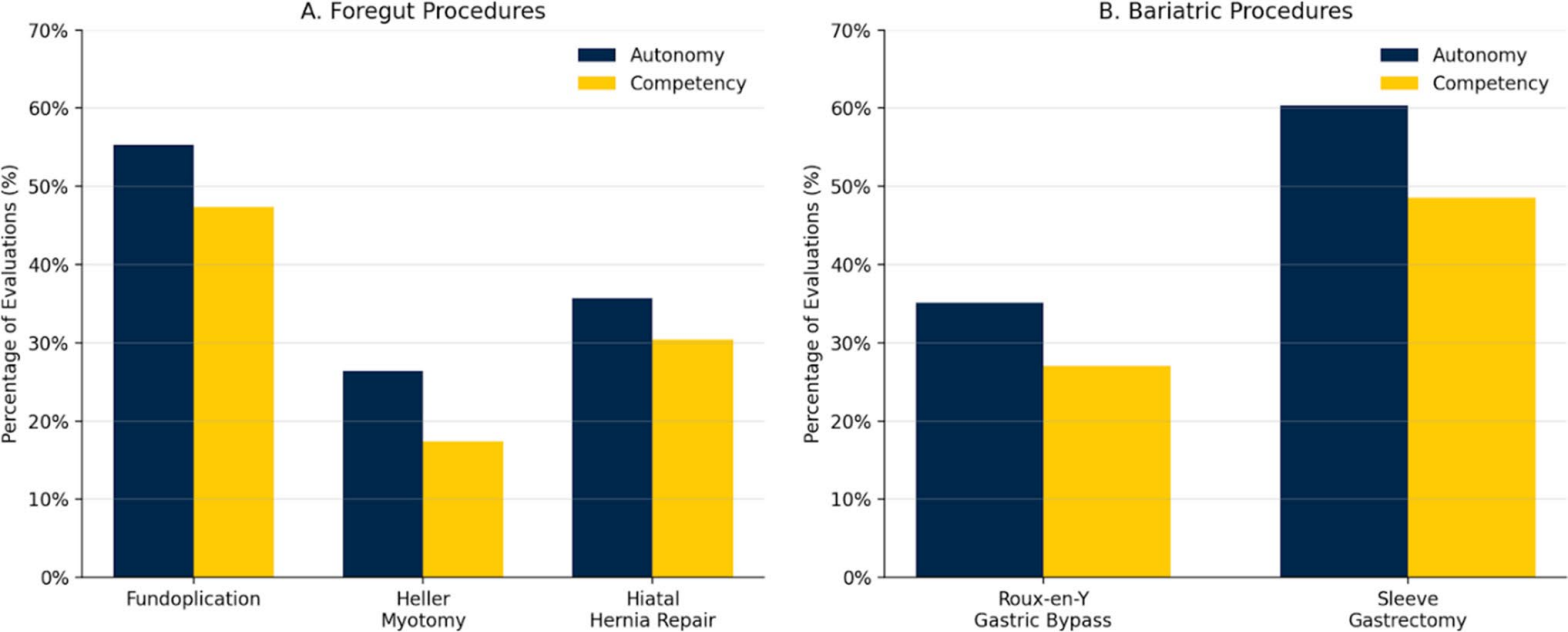
Attending rated autonomy and competency by procedure type. **A** Foregut procedures. Gastric fundoplication demonstrated the highest levels of attending-rated autonomy and competency, whereas Heller myotomy demonstrated the lowest levels among foregut procedures, **B** Bariatric procedures. Sleeve gastrectomy demonstrated higher autonomy and competency compared with Roux-en-Y gastric bypass

Within the chosen foregut procedures, there was significant variation in procedure-level assessments of performance and autonomy. Residents demonstrated the highest autonomy and competency during gastric fundoplication (respectively, 55.3% and 47.4%, *p* < 0.001) (Fig. 2A).

There was also significant variation within the included bariatric procedures with residents demonstrating the highest autonomy and competency during sleeve gastrectomies (respectively, 50.7% and 48.6%, *p* < 0.001) (Fig. 2B).

### Resident-rated autonomy and competency

Residents rated bariatric procedures higher than foregut procedures for competency and autonomy. Resident-rated autonomy was 41.6% for bariatric procedures and 33.1% for foregut procedures (*p* < 0.001). Resident-rated competency was 25.5% for bariatric procedures and 19.4% for foregut procedures (*p* < 0.001) Table 2.

**Table 2:** Attending and resident ratings of operative autonomy and competency by procedure type

		Foregut			Bariatric	
Outcome	Evaluator	Fundoplication	Heller myotomy	Hiatal hernia	RYGB	Sleeve gastrectomy
Autonomy	Attending	55.3%	26.4%	35.7%	35.1%	60.4%
	Resident	44.7%	13.2%	32.9%	21%	54.4%
Competency	Attending	47.4%	17.4%	30.4%	27.1%	48.6%
	Resident	19.4%	0%	16.2%	11.4%	33.8%

Values represent the percentage of evaluations in which residents were rated as achieving meaningful autonomy or competent performance

### Agreement between resident and attending ratings

Across all procedures, residents consistently rated operative autonomy and competency lower than attendings. Overall, attendings rated residents as autonomous in 46.2% of evaluations compared with 38.5% by residents (*p* < 0.001). Similarly, attendings rated residents as competent in 35.8% of evaluations compared with 20.6% by residents (*p* < 0.001) (Table 3).

**Table 3:** Comparison of attending and resident ratings of operative autonomy and competency

Outcome	Foregut	Bariatric	*p* value
Autonomy			
Attending rating	237 (38.2%)	545 (50.7%)	< 0.001
Resident rating	205 (33.1%)	447 (41.6%)	< 0.001
Competency			
Attending rating	188 (32.4%)	419 (40.7%)	< 0.001
Resident rating	120 (19.4%)	274 (25.5%)	< 0.001

Comparison of attending and resident ratings of operative autonomy and competency for foregut and bariatric procedures. Values are presented as the number (percentage) of evaluations classified as autonomous or competent

Procedure-level analysis demonstrated significant differences between resident and attending assessments across operations (*p* < 0.001). Residents rated their own autonomy and competency lower than their attendings across all procedure types. Gastric fundoplication had the greatest discordance in competency ratings between residents and attendings (19.4% vs 47.4%). RYGB had the greatest discordance in autonomy ratings between residents and attendings (21% vs 35.1%), while hiatal hernia repair evaluations showed the closest concordance between residents and attendings (32.9% vs 35.7%).

### Predicted probabilities of autonomy and competency

Multilevel logistic regression models were used to estimate predictors of competency and autonomy. Predicted probabilities represent the estimated likelihood of a resident being rated competent or autonomous in a typical case after accounting for model covariates.

#### Competency

In foregut procedures, significant predictors of competent performance included procedure type and residency program. Residency program accounted for 27.2% (95% CI 12.0–50.8%) of the variation in competency ratings, corresponding to an intraclass correlation coefficient (ICC) of 0.27. Competency did not vary by case complexity or timing in the academic year. The predicted probability of competent performance was 34.4% (95% CI 27.6–41.2%) (Table 4).

**Table 4:** Predicted probabilities of resident autonomy and competency

Outcome	Procedure category	Predicted probability	95% CI	Between-program variation
Competency	Foregut	34.4%	27.6–41.2%	27.2% (95% CI 12.0–50.8%)
	Bariatric	38.5%	31.9–45.2%	29.0% (95% CI 16.9–45.0%)
Autonomy	Foregut	44.6%	37.6–51.7%	32.5% (95% CI 17.5–52.3%)
	Bariatric	50.7%	44.0–57.4%	32.4% (95% CI 20.5–47.1%)

Predicted probabilities of resident competency and autonomy from multilevel logistic regression models adjusting for procedure type, residency program, case complexity, and timing within the academic year. Between-program variation represents the proportion of outcome variation attributable to differences between residency training programs

In bariatric procedures, procedure type and residency program were also predictors of competent performance. Residency program accounted for 29.0% (95% CI 16.9–45.0%) of the variation in competency, corresponding to an ICC of 0.29. Competency did not vary by case complexity or timing in the academic year. The predicted probability of competent performance was 38.5% (95% CI 31.9–45.2%) (Table 4).

#### Autonomy

In foregut procedures, predictors of meaningful autonomy included procedure type, timing in the academic year, and residency program. 32.5% (95% CI 17.5–52.3%) of variation in autonomy was associated with the residency program, corresponding to an ICC of 0.33. Meaningful autonomy did not vary by case complexity. The predicted probability of autonomous performance was 44.6% (95% CI 37.6–51.7%) (Table 4).

In bariatric procedures, predictors of meaningful autonomy included procedure type and residency program. 32.4% (95% CI 20.5–47.1%) of variation in autonomy was associated with the residency program, corresponding to an ICC of 0.32. Meaningful autonomy did not vary by case complexity, or timing in the academic year. The predicted probability of autonomous performance was 50.7% (95% CI 44.0–57.4%) (Table 4).

## Discussion

In this national analysis of SIMPL evaluations of advanced foregut and bariatric procedures, we identified four key findings. First, less than half of evaluations of chief resident performance demonstrated meaningful autonomy and competency at the end of general surgery training for both foregut and bariatric procedures. Second, overall readiness differed by procedure category with foregut cases demonstrating lower autonomy and competency compared to bariatric cases. Procedure-level analyses also demonstrated variability in readiness across operations, with higher competency and autonomy observed in higher volume procedures such as sleeve gastrectomy and fundoplication compared to other procedures. Third, residents consistently rated their competency and autonomy lower than attending surgeons across procedure types. Finally, this analysis revealed significant program-level variation in performance with approximately one-third of the variability in competency and autonomy attributable to differences between residency programs. Collectively, these findings suggest that a substantial proportion of graduating residents may not be achieving practice-ready performance and highlight the need to better define and standardize expectations for readiness for independent practice in complex minimally invasive surgery.

The readiness gaps observed in this study are consistent with broader concerns that modern training structures may unintentionally limit the continuity, autonomy, and breadth required for preparedness in complex procedures. At the same time, operative training has continued to evolve in ways that cannot be fully explained by total case volume alone. For instance, despite increases in median laparoscopic operative volume since 2005, Malangoni et al. found that many essential common operations were still infrequently performed with approximately 34% completed a median of fewer than five times during residency and several with a median frequency of zero [13]. Consistent with these concerns, nearly one-quarter of graduating general surgery residents believe that the current curriculum has not prepared them for independent practice [14]. Our study extends this literature by demonstrating minimally invasive surgery procedure-specific readiness gaps at the chief resident level using a national competency-based evaluation system.

The differences observed in readiness between foregut and bariatric procedures in our cohort may reflect variability in technical complexity, case heterogeneity, and differences in training exposure between general surgery residencies. Advanced foregut procedures may be performed less frequently and may involve greater variability in operative approach or patient factors, potentially limiting opportunities for progressive autonomy. Bariatric surgery, in contrast, may be performed within more structured institutional frameworks due to national accreditation requirements that influence case volume and operative workflows which may help explain the higher autonomy and competency observed in these cases. Interestingly, procedure-level analyses demonstrated higher competency and autonomy for sleeve gastrectomy and fundoplication, which may reflect the more standardized operative steps in these procedures as well as more opportunity for repetition given their higher case volume. Recent analyses of Fellowship Council case logs have begun to define procedural benchmarks for foregut fellowship training, reflecting increasing interest in more structured post-residency training pathways for these procedures [15]. The lower readiness observed for foregut operations in this study may help contextualize these discussions and highlight the potential role of structured training pathways in supporting procedural competency.

Importantly, suggesting that all graduating residents are expected to be fully practice ready in these operations is beyond the scope of our findings, but these results do highlight variability in operative exposure and performance in procedures that residents may still encounter in practice. While bariatric surgery benefits from more structured post-residency training pathways and credentialing requirements, foregut surgery lacks clearly defined competency benchmarks across general surgery residency, minimally invasive surgery fellowship training, and credentialing requirements, highlighting areas that need further study.

Interestingly, residents consistently rated their autonomy and competency lower than attendings across all procedures. This discordance may reflect conservative self-assessment, differing thresholds for what constitutes “practice readiness,” or rater calibration differences across institutions and faculty. Prior work describing the SIMPL assessment framework has highlighted the importance of shared expectations and rater calibration when interpreting operative performance evaluations [16, 17]. By directly comparing these perceptions using the same assessment tool, our study adds national evidence that both procedure type and evaluator perspective shape practice readiness, underscoring the importance of continued, personalized calibration in competency assessment.

A final and important finding in this analysis was the substantial variation in evaluations of operative competency and autonomy attributable to the residency program. Approximately, one-third of the variability in performance ratings was explained by program, being the first study to suggest that the training environment may meaningfully influence assessment of procedural readiness for complex procedures. Differences in operative volume, faculty supervision practices, subspecialty exposure, and institutional culture surrounding graduated autonomy may all contribute to this variability. Prior work has demonstrated that intraoperative teaching style and faculty approaches to graduated responsibility influence resident autonomy and performance development [18, 19]. Our findings extend this literature by suggesting that such differences may translate into measurable variation in readiness for independent practice across training programs for complex bariatric and foregut procedures.

Notably, procedural case complexity was not associated with competency or autonomy in our multivariable models, suggesting that differences in readiness are unlikely to be explained solely by case difficulty and instead could reflect structural differences in training opportunities and autonomy progression across programs. These findings may serve as a call for programs to more closely evaluate their internal training structures and expectations for operative independence. Program directors may benefit from examining how operative exposure, autonomy progression, and competency benchmarks for complex minimally invasive procedures are defined within their own institutions. Engagement with competency-based tools like SIMPL may help programs identify procedure-specific training gaps that warrant targeted attention.

This study has several limitations. First, SIMPL participation is voluntary and not universal, introducing potential selection bias if participating institutions differ in operative volume, case variety, educational culture, or utilization of structured competency-based assessment platforms. In addition, not all operative cases are submitted for evaluation within the SIMPL platform, and case selection may be influenced by user behavior or institutional practices rather than a standardized sampling process. Prior literature has described selective evaluation submission behaviors among trainees, particularly when assessments are perceived as summative rather than formative [20]. Conversely, some residency programs may require increased evaluation completion for trainees requiring additional oversight or remediation. As a result, the direction of this potential selection bias, whether evaluations disproportionately reflect higher or lower performing trainees, remains unclear and cannot be determined within the structure of the SIMPL dataset alone. As a result, evaluated cases may not fully represent the overall operative experience or performance of residents.

Second, SIMPL evaluations are inherently subjective, potentially influenced by rater bias, institutional norms, and differences in faculty assessment practices. In addition, important operative factors such as case urgency (elective vs. emergent) are not captured within the SIMPL dataset and may represent unmeasured confounders as operative autonomy and performance may differ between elective and emergent cases. Procedural comparisons may also be confounded by unmeasured factors such as patient complexity and attending subspecialty expertise. Interpretation of the high proportion of open foregut procedures is limited by the SIMPL database, which does not distinguish planned open operations from cases converted from minimally invasive approaches.

Third, although restricting analyses to PGY-5 residents reduced confounding by differing training levels, evaluations were collected throughout the chief resident year and therefore may reflect different stages of resident progression rather than exclusively end-of-training performance. In addition, the cross-sectional structure of the dataset limited our ability to assess individual learning curves or longitudinal progression in competency and autonomy over time.

Finally, less frequently performed procedures such as Heller myotomy may be more susceptible to variability due to smaller sample sizes. Additionally, the limited and variable number of evaluations per trainee prevented robust trainee-level analyses examining whether individual residents achieved competency or autonomy on at least one assessment. Despite these limitations, the large multi-institutional sample and use of a standardized competency-based assessment framework through SIMPL strengthen the validity of our findings.

These findings have important implications for surgical education. Ensuring that graduating residents demonstrate readiness for complex minimally invasive procedures may require greater emphasis on competency-based assessment rather than relying solely on procedural exposure during training. Competency-based assessment tools such as SIMPL may help programs identify procedure-specific gaps in training earlier, guide targeted intraoperative coaching, and intentionally structure resident autonomy progression for complex operations like those in advanced foregut and bariatric surgery. The observed resident-attending rating discordance also highlights the need for improved calibration and shared definitions of practice-ready performance by the completion of residency. Given the significant program-level variation in readiness identified in this study, national competency-based datasets may help inform future refinements in training expectations and assessment strategies for complex minimally invasive surgery. Lastly, in the context of the lower readiness observed for foregut procedures, these findings may also contribute to ongoing discussions regarding the development of more structured training pathways for foregut surgery.

## Conclusion

Evaluations of chief resident performance demonstrated limited readiness for independent practice in advanced foregut and bariatric procedures, with foregut operations showing lower competency and autonomy ratings. These findings underscore that operative volume alone is not an appropriate surrogate for practice readiness and that preparedness varies by procedure type and evaluator perspective. Competency-based assessment tools such as SIMPL offer a practical approach to identify procedure-specific gaps in surgical education and align operative experience to address these needs prior to graduation from surgical residency.
